# Efficacy of green jackfruit flour as a medical nutrition therapy replacing rice or wheat in patients with type 2 diabetes mellitus: a randomized, double-blind, placebo-controlled study

**DOI:** 10.1038/s41387-021-00161-4

**Published:** 2021-06-14

**Authors:** A. Gopal Rao, K. Sunil Naik, A. G. Unnikrishnan, James Joseph

**Affiliations:** 1 Department of Medicine, Government Medical College and Government General Hospital, Srikakulam, Andhra Pradesh India; 2 Chellaram Diabetes Institute, Pune, Maharashtra India; 3 God’s Own Food Solutions Pvt Ltd, Kochi, Kerala India

**Keywords:** Nutrition, Type 2 diabetes, Dietary carbohydrates, Randomized controlled trials, Lifestyle modification

## Abstract

**Background/Objectives:**

Medical nutrition therapy along with pharmacological interventions as a multidisciplinary approach is required to treat type 2 diabetes mellitus (T2DM). This study evaluated the efficacy of Jackfruit365™ green jackfruit flour as an integral part of daily meal in patients with T2DM.

**Subjects/Methods:**

This was a randomized, double-blind, placebo-controlled study conducted between May 2019 and February 2020. Patients of either sex aged ≥18 to ≤60 years with a diagnosis of T2DM for >1 year receiving oral antihyperglycemic agents were randomized (1:1) to receive either jackfruit flour 30 g/day (Group A) or placebo flour (Group B) (breakfast and dinner) daily for 12 weeks replacing an equal volume of rice or wheat flour. The primary endpoint was a mean change in glycosylated hemoglobin (HbA1c). Other endpoints were mean changes in fasting plasma glucose (FPG), postprandial plasma glucose (PPG), lipid profile, and body weight. The independent *t*-test was used to compare changes between the groups.

**Results:**

A total of 40 patients were enrolled (*n* = 20 each). A significantly higher reduction in HbA1c was observed in Group A compared to Group B from baseline to week 12 [−2.73 mmol/mol (−0.25%) vs. 0.22 mmol/mol (0.02%), *p* = 0.006]. The mean change in FPG and PPG was significantly higher in Group A than that of Group B (*p* = 0.043 and *p* = 0.001). The continuous glucose monitoring showed decreasing mean blood glucose in 7 days of administration of jackfruit flour meal.

**Conclusion:**

Patients from Group A had a significantly higher reduction in HbA1c, FPG, and PPG than Group B demonstrating the efficacy of jackfruit flour in glycemic control as medical nutrition therapy replacing an equal volume of rice or wheat flour in daily meal.

**Clinical trial registry:**

CTRI/2019/05/019417.

## Introduction

Type 2 diabetes mellitus (T2DM) is a chronic metabolic disorder and if left untreated, leads to uncontrolled hyperglycemia. It is reported that nearly 100 million people in India would be affected by this disease by 2030 [1]. There are various approaches available to alleviate and treat diabetes and use of medical nutrition therapy (MNT) is considered an important one. Antidiabetic medication along with MNT plays a synergistic role in the management of blood glucose level.

*Artocarpus heterophyllus*, commonly known as jackfruit, belongs to the mulberry family (Moraceae). It is mostly cultivated in the tropical regions of the world especially in the Western Ghats of India. It is rich in dietary fiber, carbohydrates, vitamins, and minerals. The green or unripe jackfruit is used as a vegetable. The ripe fruit is very sweet and rich in sugars. There are many concerns surrounding jackfruit consumption by patients with diabetes mainly because of this sweet taste when ripe.

Carotenoids, proanthocyanidin, flavonoids, volatile acids, sterols, and tannins are some of the phytochemical compounds present in the jackfruit [2–5]. The antidiabetic effect of jackfruit has been reported in several in vitro and in vivo studies [6–11]. That could be due to high proanthocyanidin and flavonoids [12, 13]. The possible mechanism of action is inhibition of lipid peroxide formation reported by Biworo et al. [6].

Assessment and evaluation of the nutritional and glycemic value of green jackfruit as an alternative to rice for diabetes in Kerala demonstrated a reduction in the demand for antidiabetic medication during jackfruit season [14]. To our knowledge, no randomized controlled trial has been conducted to evaluate the efficacy of green jackfruit flour to achieve optimal glycemic control in patients with T2DM. This study aimed to evaluate the efficacy of Jackfruit365™ green jackfruit flour in patients with T2DM.

## Subjects and methods

### Trial design

This was a randomized, double-blind (participants and investigator), placebo-controlled study (CTRI/2019/05/019417) conducted at Rajiv Gandhi Institute of Medical Sciences, Srikakulam, Andhra Pradesh, India, between May 2019 and February 2020. The study protocol was reviewed and approved by the Institutional Ethics Committee. The study was conducted in accordance with the Declaration of Helsinki and International Conference on Harmonization guidelines for Good Clinical Practice. Written informed consent was obtained from all patients prior to their enrolment into the study.

Eligible patients were randomized using a computer-based, predetermined randomization technique (1:1; simple randomized, sequentially numbered, sealed, opaque envelopes) into two groups. Study group to receive 30 g of Jackfruit365™ green jackfruit flour (Group A): 15 g (equivalent to 22.5 mL) each using a measuring spoon added to breakfast and dinner meals prepared from rice batter or wheat flour, respectively. Control group to receive placebo flour (Group B): of an equal measure of rice flour and wheat flour for breakfast and dinner, respectively, to match their diet before intervention. Both groups add jackfruit flour or placebo flour first to the container and then add regular batter flour to the same level as normal portion size. With the portion size the same as before intervention, both jackfruit flour and placebo flour make an equal volume replacement of rice or wheat flour. Randomization was done at the CRO site. A subset of five patients from each group also underwent a masked continuous glucose monitoring (CGM) for 14 days (7 days after screening and 7 days after randomization) using FreeStyle Libre Pro. The maximum period of study participation for any patient was 13 weeks (1 week of screening and 12 weeks of double-blind treatment).

Green jackfruit flour is prepared from dehydrated, mature, unripe jackfruits. Nutritionally, 30 g of green jackfruit flour has calorie of 108 kcal, net carbohydrates of 20 g, and dietary and soluble fiber content of 4 and 1 g. This was compared to equal volume of placebo flour, which contains 146 calories, 30 g of net carbohydrates, 2.54 g of dietary fiber, and 0 g of soluble fiber.

A detailed medical history was obtained at the screening visit from patients. Primary clinical examination included evaluation of HbA1c, while secondary examinations were FPG, PPG, body weight, body mass index (BMI), and CGM.

### Participants

Patients of either sex, aged ≥ 18 and ≤60 years, diagnosed with T2DM for >1 year, stabilized on the oral hypoglycemic agent(s) (monotherapy or polytherapy), and having HbA1c value <8% at screening were eligible for the study. Patients under other concomitant medications, in addition to oral hypoglycemic agents, were also included in this study.

Pregnancy was an exclusion criterion. Patients with T2DM who were on insulin, had known cases of severe or chronic hepatic or renal disease or any active malignancy, had a history of a significant cardiovascular event < 12 weeks prior to randomization, had a history of known major complications of diabetes such as ketoacidosis, nephropathy, neuropathy, retinopathy, and diabetic wounds or had a history of known chronic, contagious infectious disease, such as active tuberculosis, hepatitis B or C, or HIV, active metabolic or gastrointestinal diseases that may interfere with nutrient absorption, metabolism, or excretion, excluding diabetes, and patients using any other investigational drug within 1 month prior to recruitment or who were at that time participating in any other clinical study were excluded from this study.

### Outcomes

The primary efficacy endpoint was mean change from baseline in HbA1c levels at week 12. Secondary endpoints included mean change from baseline in FPG, PPG, body weight, and lipid profile at week 12 and change in glucose level by CGM for 2 weeks.

### Statistical analysis

Data were analyzed using the Statistical Analysis System (SAS^®^) for windows version 9.4. Qualitative data were presented as numbers and percentages, while quantitative data were presented as mean (standard deviation [SD]). A comparison of quantitative variables between the groups was done using the independent *t*-test. *p* < 0.05 was considered statistically significant.

## Results

**Fig. 1:**
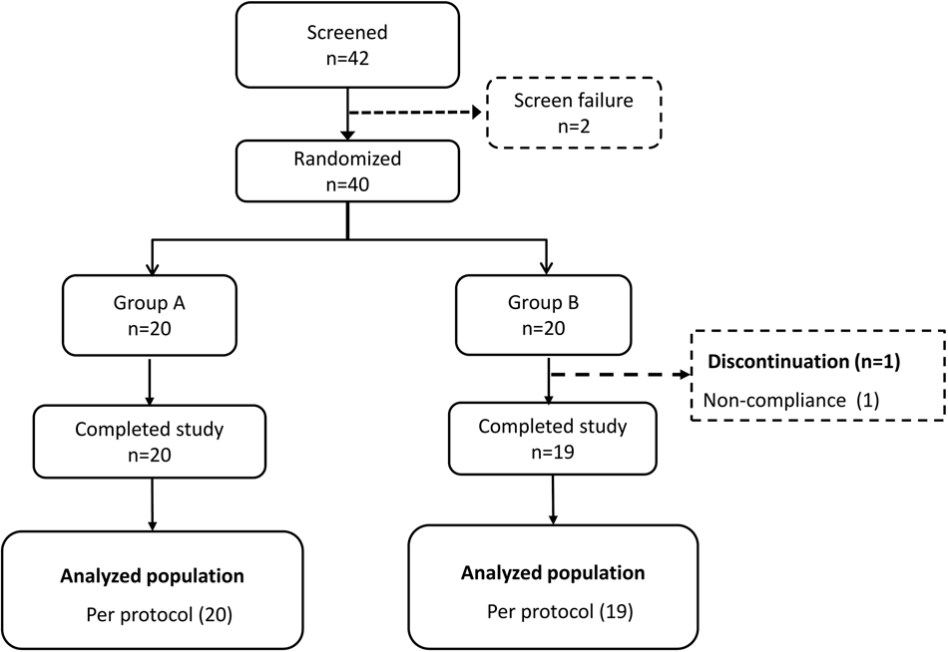
CONSORT diagram. Group A, received green jackfruit flour meal. Group B, received placebo meal.

A total of 42 patients were enrolled; of which, 2 subjects were excluded as not eligible for the clinical study and 40 patients were randomized into two groups to receive green jackfruit flour (Group A, *n* = 20) or placebo flour (Group B, *n* = 20). One patient from the placebo group was lost to follow up during the study (Fig. 1).

The patients’ demographic characteristics are summarized in Table 1. The mean ages of patients in Group A and Group B were 46.15 years and 46.10 years, respectively. In Group A and Group B, the prevalence of men was higher (Table 1). Glimepiride/metformin was the most common concomitant medication received (Group A, 40% and Group B, 65%).

**Table 1:** Baseline characteristics: the study subjects^a^.

Variables	Group A (*n* = 20)	Group B (*n* = 20)
Age (years)	46.15 (9.66)	46.10 (8.68)
Sex, *n* (%)		
Men	12 (60)	13 (65)
Women	8 (40)	7 (35)
BMI (kg/m^2^)	24.73 (1.90)	23.94 (1.79)
Height (cm)	160.75 (8.53)	160.15 (7.98)
Weight (kg)	64.85 (8.19)	62.15 (7.17)
Duration of diabetes (years)	5.90 (4.45)	5.77 (3.88)
Concomitant medication		
Amlodipine	1 (5)	2 (10)
Cetirizine	1 (5)	0
Diclofenac/linseed oil/menthol/methyl salicylate	1 (5)	0
Glibenclamide	1 (5)	0
Glimepiride	6 (30)	5 (25)
Glibenclamide/metformin	2 (10)	1 (5)
Glyburide/metformin	0	1 (5)
Sitagliptin/metformin	0	1 (5)
Sitagliptin	1 (5)	0
Glimepiride/metformin	3 (15)	1 (5)
Metformin	8 (40)	13 (65)
Paracetamol	2 (10)	0
Sitagliptin	4 (20)	4 (20)
Telmisartan	1(5)	0
Vildagliptin	0	1(5)

Data are shown as mean (SD).

*BMI* body mass index.

^a^Changes in glycemic and lipid parameters are described in subsequent tables.

### Primary endpoint

**Fig. 2:**
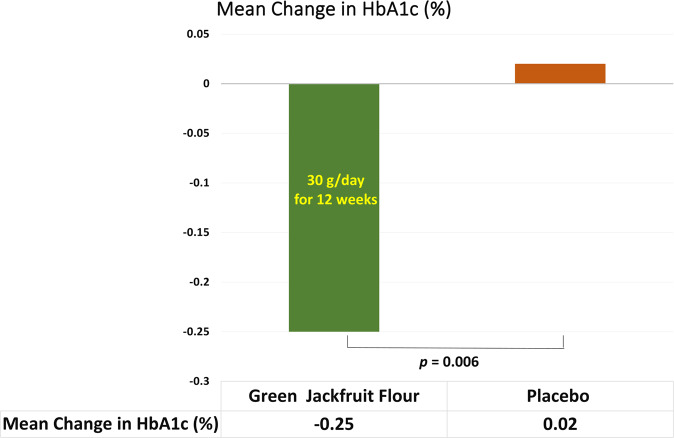
Mean change in HbA1c from baseline to week 12 across both groups.

The mean HbA1c level was reduced from baseline 55.57 mmol/mol (7.23%) to week 12 52.84 mmol/mol (6.98%) with the mean change of −2.73 mmol/mol (−0.25%) in Group A (Fig. 2). In Group B, the mean HbA1c level at baseline was 55.88 mmol/mol (7.26%) and at 12 weeks was 56.11 mmol/mol (7.28%). At the end of 12 weeks, the mean HbA1c did not decline in patients who received placebo flour and the mean difference observed was an increase of 0.22 mmol/mol (0.02%). A significant decrease in HbA1c was observed in the participants of Group A demonstrating the superiority of Group A over Group B (−0.25% vs. 0.02%; *p* = 0.006) (Table 2).

**Table 2:** Change from baseline to week 12 visit in the levels of HbA1c, FPG, PPG, and body weight.

Parameters	Timepoints	Group A (*n* = 20)	Group B (*n* = 19)	*p* value
Primary endpoint				
HbA1c (mmol/mol)	Baseline	55.57 (5.17)	55.88 (4.95)	0.850
	Week 12	52.84 (5.20)	56.11 (4.69)	0.046
Mean change from baseline		−2.73	0.22	0.006
HbA1c (%)	Baseline	7.23 (0.47)	7.26 (0.45)	0.851
	Week 12	6.98 (0.48)	7.28 (0.49)	0.046
Mean change from baseline		−0.25	0.02	0.006
Secondary endpoints				
FPG (mg/dL)	Baseline	142.85 (22.16)	146.73 (13.32)	0.513
	Week 12	113.40 (10.37)	130.57 (8.78)	0.001
Mean change from baseline		−29.45	−16.15	0.043
FPG (mmol/L)	Baseline	7.93 (1.23)	8.14 (0.74)	0.514
	Week 12	6.29 (0.57)	7.25 (0.48)	0.001
Mean change from baseline		−1.63	−0.89	0.043
PPG (mg/dL)	Baseline	199.35 (21.08)	196.52 (13.84)	0.625
	Week 12	162.65 (10.80)	187.89 (14.98)	0.001
Mean change from baseline		−36.70	−8.63	0.001
PPG (mmol/L)	Baseline	11.07 (1.17)	10.91 (0.77)	0.628
	Week 12	9.03 (0.60)	10.43 (0.83)	0.001
Mean change from baseline		−2.03	−0.47 (0.88)	0.001
Body weight (kg)	Baseline	64.85 (8.19)	62.15 (7.17)	0.14
	Week 12	65.02 (8.26)	62.10 (7.26)	0.12
Mean change from baseline		−0.17	0.05	0.20

Data are represented as mean (SD).

*HbA1c* glycemic control, *FPG* fasting blood glucose, *PPG* postprandial plasma glucose.

### Secondary endpoints

In Group A, the mean FPG at baseline was 7.93 mmol/L (142.85 mg/dL) and at the end of the study (12 weeks), it was 6.29 mmol/L (113.40 mg/dL). The mean FPG level was decreased in the patients of Group A from baseline to week 12 with the mean change of −1.63 mmol/L (−29.45 mg/dL). In Group B, the mean FPG at baseline was 8.14 mmol/L (146.73 mg/dL) and at week 12 was 7.25 mmol/L (130.57 mg/dL). At the end of 12 weeks, the mean FPG declined in patients from Group B, and the mean difference observed was −0.89 mmol/L (−16.15 mg/dL).

The mean FPG level was significantly lower in the Group A compared to Group B at week 12 [6.29 mmol/L (113.40 mg/dL) and 7.25 mmol/L (130.57 mg/dL), *p* = 0.001)]. Also, the mean change in the FPG level was significantly higher in the patients from Group A compared to Group B (*p* = 0.043).

There was a reduction in the mean PPG level at week 12 in the patients from Group A [at baseline: 11.07 mmol/L (199.35 mg/dL) and at week 12: 9.03 mmol/L (162.65 mg/dL)]. The mean change from baseline to week 12 was −2.03 mmol/L (−36.70 mg/dL) in the patients from Group A. In Group B, the mean PPG level at baseline was 10.91 mmol/L (196.52 mg/dL) which was reduced to 10.43 mmol/L (187.89 mg/dL) at week 12 and the mean change was −0.47 mmol/L (−8.63 mg/dL). A significant decrease in PPG level was observed in the patients of Group A compared to Group B (*p* = 0.001). There were no significant changes in the weight of the participants of both the groups observed during the study. The lipid profile of patients in Group A and Group B is summarized in Table 3. The levels of cholesterol, HDL, LDL, and triglycerides remained unchanged from baseline to week 12, and these levels are comparable between both the groups.

**Table 3:** Lipid profile.

Parameters	Timepoints	Group A (*n* = 20)	Group B (*n* = 19)
Cholesterol (mg/dL)	Baseline	184.1 (40.31)	181.2 (41.03)
	Week 12	178.1 (23.29)	182.35 (26.24)
HDL (mg/dL)	Baseline	40.05 (6.59)	39.45 (6.47)
	Week 12	43.70 (5.96)	43.5 (5.87)
LDL (mg/dL)	Baseline	131.6 (10.83)	138.7 (15.05)
	Week 12	137.3 (9.34)	141.25 (12.58)
Triglycerides (mg/dL)	Baseline	134.45 (16.68)	136.25 (16.17)
	Week 12	132.55 (15.25)	139.05 (13.35)

Data shown as mean (SD).

*HDL* high-density lipoprotein, *LDL* low-density lipoprotein.

The CGM data of both Group A and B showed numerically lower glucose levels over a period of 7 days following randomization (Table 4). One of the patients from Group B removed the CGM probe early and another patient was lost to follow-up, hence only three patients were evaluable. There were 7 days of monitoring before and after initiation of jackfruit flour.

**Table 4:** Average blood glucose level in continuous glucose monitoring.

Patient number	Average blood glucose (mg/dL)	
	Before start of IP	After start of IP
Participants taking jackfruit flour		
1	125.71	107.37
2	102.85	90.25
3	330.14	174.62
4	317.57	206.75
5	218.28	233.25
Mean (SD)	218.91 (105.19)	162.45 (61.98)
Mean difference between before and after start of IP	56.46	
Participants taking placebo		
6	209.57	172.75
7	339.71	200.00
8	220.28	178.12
Mean (SD)	256.52 (72.24)	183.62 (14.43)
Mean difference between before and after start of IP	72.49	

Data are represented as mean, unless otherwise specified.

Change in dose of oral hypoglycemic agents was not reported by any of the participants throughout the study duration. Patients did not report any hypoglycemia episodes during the study.

## Discussion

Diabetes is a serious health concern with multiple risk factors and many treatments are available for management. Recently, MNT has been recommended by nutritionists as a primary therapy for the management of diabetes. The goals of nutrition therapy include promoting and supporting healthful eating patterns, maintaining the pleasure of eating while addressing individual nutrition needs. The success of MNT depends on patient adherence to the dietary pattern to achieve overall goals. A randomized cross-over study involving patients with diabetes receiving MNT along with antidiabetic medication reported significant reduction (1–2%) in HbA1c levels and other outcomes with improved quality of life compared with patients receiving only antidiabetic treatment [15].

Several benefits of MNT have been attributed to improving or maintaining glycemic targets. The American Diabetic Association 2019 recommends that patients with T2DM should consume a lower number of calories and carbohydrates and a higher amount of dietary fibers to improve glycemic control [16]. In addition, the evidence suggests that high fiber intake with low calorie consumption may reduce HbA1c in patients with T2DM [17]. In the present study, the jackfruit flour had 25% lower calories, 33% lower net carbohydrates, and 57% more fiber in comparison to placebo flour suggesting that green jackfruit flour may be a promising intervention to improve glycemic control as a replacement to rice or wheat flour.

The present study carried out the effectiveness of green jackfruit when integrated into daily meals using the product of jackfruit flour as MNT in patients with T2DM. The key findings suggest that replacing an equal volume of rice or wheat flour in daily meals with jackfruit flour is significantly effective at lowering the plasma glucose level in patients with T2DM. The mean reduction in HbA1c at week 12 was significantly higher in patients supplemented with jackfruit flour, while in patients supplemented with placebo flour have reported a slight increase in HbA1c at week 12 (*p* = 0.006). Similarly, the mean FPG and PPG level was significantly lower in patients supplemented with jackfruit flour at week 12 (*p* = 0.001). The CGM in patients who were taking jackfruit flour showed decreased blood glucose levels. However, the CGM showed a significant decrease in blood glucose levels in both groups which could be attributed to the combined placebo effect of CGM and the intervention, which was not seen in the full duration of the study on HbA1c. In addition, these results may be inconclusive as two of the five patients (40% dropout) with CGM from the placebo group were excluded from the analysis.

Preclinical studies have been conducted to evaluate the efficacy of antidiabetic activity of jackfruit extract in diabetic rats. The ethanol extract of jackfruit seed in glucose loaded normal rats showed significantly increased antihyperglycemic activity compared to standard drug glibenclamide [18]. Another study showed glucose lowering effects of ethyl acetate fraction of mature leaves of jackfruit (20 mg/kg) in streptozotocin-induced diabetic rats [19]. Jain et al. reported reduction in blood glucose level and increase in serum insulin in alloxan-induced diabetic rats, along with decrease in total cholesterol and LDL and increase in body weight and HDL [20]. The glucose lowering effect could be due to the increased insulin secretion from available beta cells of Langerhans or release from bound form [20].

Previously reported studies showed that the jackfruit plant has a potential glucose lowering effect in animal models. An active component flavonoid is considered to initiate the glucose lowering effect [21, 22]; however, β carotene has an antioxidant property which inhibits lipid peroxidation [6]. An interventional single-blinded clinical study evaluated the glucose lowering effect of leaf decoction of jackfruit in patients with T2DM. A significant reduction in the levels of FPG and PPG was observed after the treatment (*p* < 0.001). The leaf decoction of fruit was found to be effective for improving glucose tolerance capacity [10]. Similar antidiabetic effects were observed in the present study.

The clinical profile and pattern of diabetes is different in the Indian population compared to Western population. The prevalence of diabetes is increasing worldwide with increasing trend of overweight and obesity [23]. However, more than 30% of patients with diabetes in India reported to be nonobese [24]. The South Asian population has a higher risk of diabetes at low BMI and dietary interventions that achieve normoglycemia without weight loss may also be important [25]. In the present study, a significant reduction in HbA1c level was observed, without a significant reduction in weight. This could be due to comparatively normal mean BMI in this study and which may suggest that jackfruit flour can be useful in such a population with diabetes for improving glycemic control without weight loss.

This study was first of its kind. Strengths of the present study are that it was double-blind placebo-controlled and the diet after intervention was culturally sensitive and similar to the real-life diet. As intervention was integrated into several traditional home cooked foods, it was possible to achieve adherence to the intervention. Authors also acknowledge a smaller sample size, shorter duration for study, lower HbA1c exclusion criteria to avoid hypoglycemia, or alteration to medication and the study was conducted at a single center as limitations of the study. With the efficacy data and adherence to intervention known from this study, a multicentric multicountry powered study with a larger population, for longer duration, and higher HbA1c may be needed to further confirm clinical significance and generalize these outcomes to a wider population.

## Conclusion

Consumption of Jackfruit365™ green jackfruit flour, 30 g per day, was found to be effective in lowering the levels of HbA1c, FPG, and PPG compared to placebo flour in patients with T2DM replacing an equal volume of rice or wheat flour in daily meals. Green jackfruit is a good source of fiber, has fewer calories, and in flour form makes it easy to include in a variety of daily food like roti, porridge, or pancakes as part of MNT for T2DM without changing eating habits.

## Data Availability

The data used to support the findings of this study are included within the article.
